# COVID-19 outbreak in Italy: an opportunity to evaluate extended interval dosing of ocrelizumab in MS patients

**DOI:** 10.1007/s00415-023-12084-4

**Published:** 2023-11-20

**Authors:** Alvino Bisecco, Federica Matrone, Marco Capobianco, Giovanna De Luca, Massimo Filippi, Franco Granella, Giacomo Lus, Girolama Alessandra Marfia, Massimiliano Mirabella, Francesco Patti, Maria Trojano, Agnese Mascolo, Massimiliano Copetti, Gioacchino Tedeschi, Antonio Gallo, the OCREVID study group on behalf of the Italian MS Register, Simona Malucchi, Maria Talentacci, Valentina Tomassini, Deborah Farina, Lucia Moiola, Agostino Nozzolillo, Alessandro Franceschini, Matteo Minetti, Elisabetta Signoriello, Giuseppe Romano, Mario Risi, Alessandro d’Ambrosio, Doriana Landi, Carolina Gabri Nicoletti, Assunta Bianco, Matteo Lucchini, Clara Chisari, Simona Toscano, Damiano Paolicelli, Pietro Iaffaldano, Matilde Inglese, Maria Cellerino, Paolo Bellantonio, Roberta Fantozzi, Giuseppe Salemi, Paolo Ragonese, Maura Danni, Gabriella Coniglio, Diana Ferraro, Giorgia Teresa Maniscalco, Antonella Conte, Paola Cavalla, Marika Vianello, Daniela Cargnelutti, Maurizia Gatto, Ardito Buonaventura, Alessandra Lugaresi, Maria Pia Amato, Paola Gazzola, Rosa Iodice, Ilaria Pesci, Sara Montepietra, Carlo Pozzilli, Elisabetta Ferraro, Mauro Zaffaroni, Davide Nasuelli

**Affiliations:** 1 I Division of Neurology, Department of Advanced Medical and Surgical Sciences, University of Campania Luigi Vanvitelli, Piazza Miraglia, 2, 80138 Naples, Italy; 2 SCDO Neurology and Regional Reference Multiple Sclerosis Center, A.O.U. San Luigi, Orbassano, Italy; 3 Department of Neurology, AO S. Croce e Carle, Cuneo, Italy; 4 Multiple Sclerosis Centre, Neurology Unit, SS. Annunziata University Hospital, Chieti, Italy; 5 MS Center, Neurology Unit, IRCCS San Raffaele Scientific Institute, Milan, Italy; 6 Neuroimaging Research Unit, Division of Neuroscience, IRCCS San Raffaele Scientific Institute, Vita-Salute San Raffaele University, Milan, Italy; 7 Vita-Salute San Raffaele University, Milan, Italy; 8 Unit of Neurosciences, Department of Medicine and Surgery, University of Parma, Parma, Italy; 9 Multiple Sclerosis Centre, Unit of Neurology, Department of General Medicine, Parma University Hospital, Parma, Italy; 10 MS Center - II Division of Neurology, Department of Advanced Medical and Surgical Sciences, University of Campania Luigi Vanvitelli, Naples, Italy; 11 Multiple Sclerosis Clinical and Research Unit, Department of Systems Medicine, Tor Vergata University and Hospital, Rome, Italy; 12 Multiple Sclerosis Center, Fondazione Policlinico Universitario “A. Gemelli” IRCCS, Rome, Italy; 13 Centro Di Ricerca Sclerosi Multipla (CERSM), Università Cattolica del Sacro Cuore, Rome, Italy; 14 Department of Medical and Surgical Sciences and Advanced Technologies, G. F. Ingrassia, University of Catania, Catania, Italy; 15 University “Aldo Moro” of Bari, Bari, Italy; 16 Unit of Biostatistics, IRCCS “Casa Sollievo della Sofferenza”, San Giovanni Rotondo, Italy

**Keywords:** MS (multiple sclerosis), COVID-19 Pandemic, Ocrelizumab, Extended interval dosing

## Abstract

**Introduction:**

During the COVID-19 pandemic, ocrelizumab (OCR) infusions for MS patients were often re-scheduled because of MS center's disruption and concerns regarding immunosuppression. The aim of the present study was to assess changes in OCR schedule during the first wave of pandemic in Italy and to evaluate the effect of delayed infusion on clinical/radiological endpoints.

**Methods:**

Data were extracted from the Italian MS Register database. Standard interval dosing was defined as an infusion interval ≤ 30 weeks, while extended interval dosing was defined as an infusion interval > 30 weeks at the time of the observation period. Clinico-demographics variables were tested as potential predictors for treatment delay. Time to first relapse and time to first MRI event were evaluated. Cumulative hazard curves were reported along their 95% confidence intervals. A final sample of one-thousand two patients with MS from 65 centers was included in the analysis: 599 pwMS were selected to evaluate the modification of OCR infusion intervals, while 717 pwRMS were selected to analyze the effect of infusion delay on clinical/MRI activity.

**Results:**

Mean interval between two OCR infusions was 28.1 weeks before pandemic compared to 30.8 weeks during the observation period, with a mean delay of 2.74 weeks (*p* < 0.001). No clinico-demographic factors emerged as predictors of infusion postponement, except for location of MS centers in the North of Italy. Clinical relapses (4 in SID, 0 in EID) and 17 MRI activity reports (4 in SID, 13 in EID) were recorded during follow-up period.

**Discussion:**

Despite the significant extension of OCR infusion interval during the first wave of pandemic in Italy, a very small incidence of clinical/radiological events was observed, thus suggesting durable efficacy of OCR, as well as the absence of rebound after its short-term suspension.

## Introduction

Ocrelizumab (OCR) is a recombinant human anti-CD20 monoclonal antibody (MAb) approved by European Medicines Agency (EMA) in 2017 [6] and by Italian Medicines Agency (AIFA) in 2018 for relapsing forms of MS (RMS) and for primary progressive MS (PMS). The maintenance infusions of OCR are given as a 600 mg IV infusion every 6 months, beginning 6 months after the starting doses [6].

Concerns emerged during COVID-19 pandemic about immunosuppressive therapies potentially exacerbating COVID-19 infection and about management of OCR and other similar high efficacy disease modifying therapies (DMTs) in patients with MS (pwMS) [18]. Although not entirely consistent, observational data suggested that anti-CD20 DMTs may increase the risk of a more severe course of COVID-19 [7, 16]. A multinational cohort study that included 1683 pwMS and confirmed COVID-19 found that the risk of hospitalization and intensive care unit admission was increased for patients taking either OCR or Rituximab, another anti-CD20 MAb [16].

In addition to the above, other reasons and premises made MS neurologists concerned about how to manage OCR-treated MS patients during COVID-19 pandemic.

First, they had to take into account that in-hospital administration of OCR represented an adjunctive risk of unintended exposure to the virus for a more fragile MS population, such that of OCR-treated MS patients, which have, on average, an older age and more severe disability compared to the general MS population (because of the indication/usual utilization of this drug).

Second, previous data showed that immune response to infections and vaccines in pwMS treated with OCR might be significantly weakened when compared with healthy controls (HCs), thus suggesting that postponement of OCR infusion might enhance the response to a possible SARS-CoV-2 infection and improve vaccine effectiveness once available [3, 5, 7].

Finally, during first COVID-19 pandemic waves, MS centers (MSc) had often to deal with a major redistribution of healthcare workforces and resources, such that it became hard to keep on with routine activities and on schedule OCR infusions. In this context, COVID-19 has represented a unique challenge for MS neurologists, especially in the countries that were most strongly affected by the pandemic, such as Italy, that was precociously and severely hit by the pandemic and had to face, at least in some areas, a dramatic and long-lasting disruption of the healthcare system. As a result, different management approaches were applied at Italian MSc as regard OCR infusion schedules during the pandemic.

MS International Federation and National MS Associations have suggested to consider postponing OCR infusion in selected stable patients, especially if B cells are depleted, at the time of the scheduled dose (https://www.nationalmssociety.org/coronavirus-covid-19-information/covid-19-vaccine-guidance) in order to (1) prevent the risk of severe illness, (2) avoid in-hospital contagion, and (3) enable patients to get a full vaccination cycle (https://www.msif.org/news/2020/02/10/the-coronavirus-and-ms-what-you-need-to-know/).

Nevertheless, it is noteworthy to consider that the concept of extended interval dose (EID) for OCR is not defined, and that there are only few efficacy and safety data in this regard [14, 15, 19, 20].

Recent reports have tried to evaluate the impact of OCR EID on clinical and radiological disease activities in pwMS during the COVID-19 pandemic. Albeit with discordant results, these studies have suggested that postponing OCR treatment could have been a reasonable and safe strategy [9, 14, 19] but have also suggested caution in terms of possible clinical/MRI reactivation [20] following CD20 B cells repopulation [15, 19]. Since these preliminary studies suffered a number of limitations, further investigations are warranted on this relevant topic.

On this background, we aimed to run a nation-wide registry-based study, in order to:

describe the consequences of the first (dramatic) wave of the COVID-19 outbreak (observation period: Feb–Jun 2020) in Italy on the schedule of OCR therapy and to investigate predictive factors determining OCR infusions delay;
2)evaluate—in patients with RMS—the possible effect of delayed OCR infusion schedule on clinical (Annualized Relapse Rate [ARR]) and radiological endpoints (new T2-hyperintense lesions and/or new T1-Gd + lesions).

Our working hypothesis was that many OCR infusion intervals were extended—(on average) for a few weeks—during the first COVID-19 outbreak in Italy, but according to OCR pharmacodynamics suggesting a long-lasting effect of the drug beyond the 6-month scheduled interval[1], the impact on MS disease—in terms of clinical and MRI activity—was small.

## Methods

We performed a retrospective, multicenter (nation-wide) observational study on a very large cohort of OCR-treated pwMS extracted from the Italian Multiple Sclerosis Registry (IMSR; supported by the Italian MS Foundation), which included core clinical data of more than 65,000 pwMS at the time of the study.

Inclusion criteria were:

- MS diagnosis,
- Age > 18 years,
- An OCR infusion scheduled during the observation period, corresponding to the first COVID-19 outbreak (and lockdown) in Italy (February-June 2020 = lock cycle, LC),
- At least one previous OCR cycle (the last cycle before the observation (lockdown) period = pre-lock cycle, PLC),
- Availability of a follow-up of at least 12 weeks after LC,
- Full reported mandatory data: demographics (age, sex), disease characteristics (MS phenotype, disease duration, Expanded Disability Status Scale—EDSS—score, number and dates of previous OCR infusions, clinical relapses), location of MSc among three Italian macro-regions (North, Center, South).

**Fig. 1:**
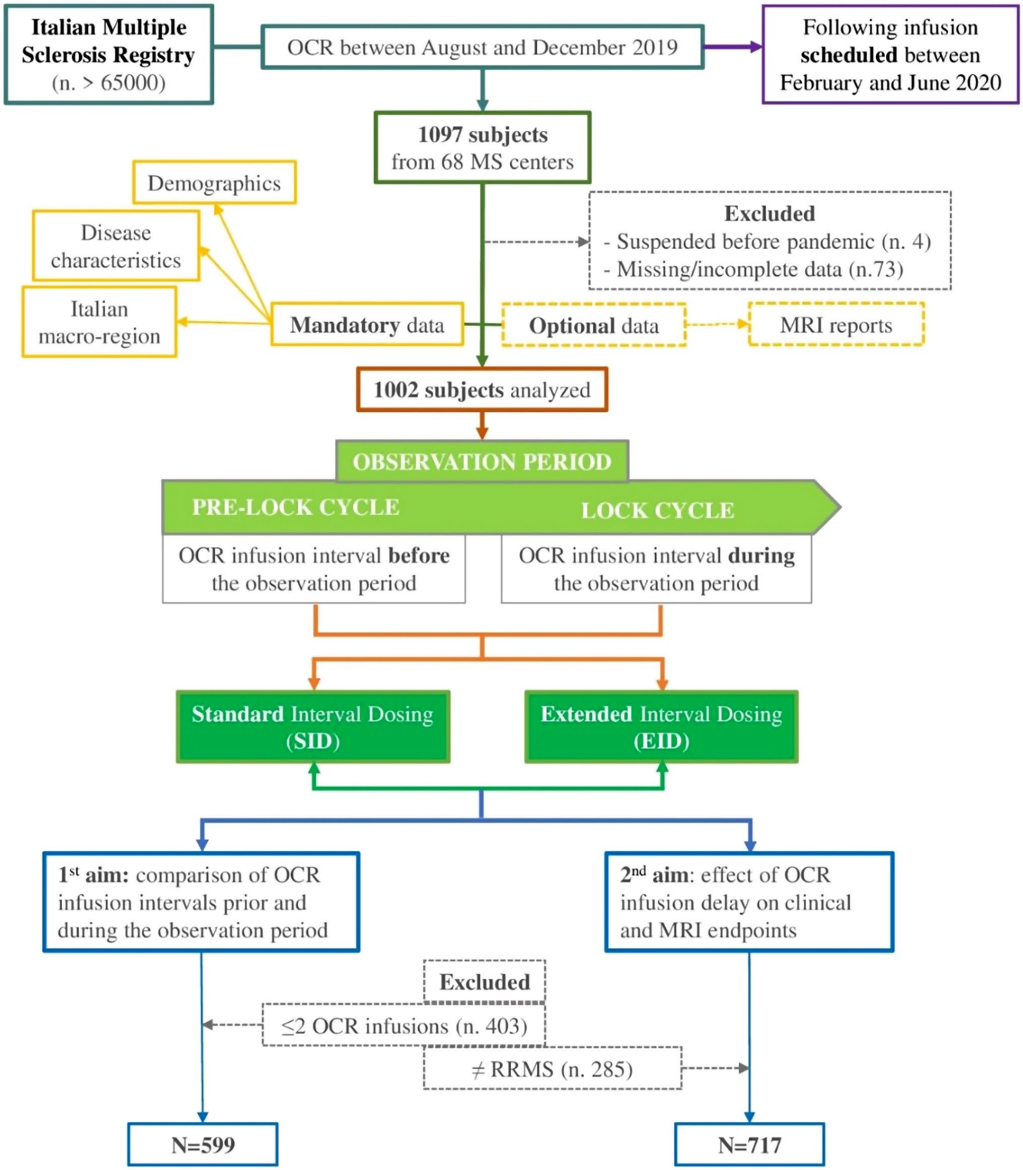
Flowchart of patients selection and analysis. *N* number, *OCR* Ocrelizumab, *MS* Multiple Sclerosis, *PRE-LOCK cycle* OCR infusion interval before the observation period, *LOCK cycle* OCR infusion interval during the observation period, *SID* standard interval dosing, *EID* extended interval dosing, *RRMS* relapsing remitting MS

We identified 1097 patients from 68 MSc with an OCR infusion between August and December 2019, such that the following OCR infusion would have been scheduled during the observation period (Fig. 1).

For the first OCR cycle (consisting of 2 × 300 mg infusion with a 2-week interval), we considered the first date reported in the IMSR.

Among the 1097 pwMS whose data were extracted from the IMSR, 4 patients suspended OCR treatment before pandemic, 1 patient was lost at follow-up and 72 further patients had missing or incomplete mandatory data and, thus, were excluded.

Possible scenarios at the time of OCR infusion at LC, according previous studies definitions [14, 15, 20], were:

1. Standard interval dosing (SID), defined as an interval between the PLC and the LC cycle ≤ 30 weeks;
2. Extended interval dosing (EID), defined as an interval > 30 weeks between PLC and LC.

Two patients with an interval between pre-lock and lock cycle ≤ 21 weeks were excluded due to excessive deviation from clinical recommendations and 16 patients with an interval dosing ≥ 52 weeks were also excluded because of the likelihood of a non-reported infusion or bridge therapy during this period.

The final sample included in statistical analyses included 1002 MS patients.

Data extracted from the IMSR were the following: demographics (age, sex), disease characteristics (MS phenotype, disease duration, Expanded Disability Status Scale—EDSS—score, number and dates of previous OCR infusions, clinical relapses), location of MSc among three Italian macro-regions (North, Center, South), MRI reports (optional data: (new T2-hyperintense lesions and/or new T1-Gd + lesions).

All participating MSc were invited via e-mail to double check uploaded data in order to sort out missing or incomplete data.

For the first aim (comparison between OCR infusion intervals prior and during the observation period and identification of predictors of delay), we considered a subgroup of 599 MS patients (those with at least two OCR infusions before the observation period: i.e., at least three OCR infusions in total), in order to have an intra-patient benchmark for OCR infusion intervals.

For the second aim (effect of delay on clinical/MRI activity), we considered the whole RMS population (*n* = 717) and excluded PMS patients, due to the shortness of the available follow-up not adequate to evaluate the effects of infusions delay on progression outcomes [8, 12].

The RMS population was prospectively observed until 12 weeks following the LC (either postponed or not), since clinical and/or radiological activity in this period might be linked to OCR infusion postponement.

Clinical and radiological activities occurring before 26 weeks after PLC and beyond 12 weeks after (on schedule or postponed) LC were considered as independent of LC timing and—for this reason—not included in the analysis.

### Statistical analysis

Patients’ baseline characteristics were reported as mean ± SD (or median and interquartile range) and frequency and percentage for continuous and categorical variables, respectively.

Group comparisons were carried out using un-paired *t*-test or ANOVA for continuous variables and Pearson χ^2^ test for categorical variables as appropriate, Bonferroni-corrected.

PLC and LC intervals dosing were compared using paired *t*-test.

Demographics disease characteristics and location of MSc among three Italian macro-regions were tested as potential predictors for treatment delay.

Potential predictors of intra-patient change in interval dosing were assessed using a linear model analysis.

Time to first relapse and time to first MRI events after PLC infusion were analyzed using Kaplan–Meier method. Cumulative hazard curves were reported along their 95% confidence intervals.

Cumulative hazard curves were compared according to the dichotomized interval dosing (≤ 30 weeks vs. > 30 weeks) using the log-rank test.

Proportional hazards Cox regression model was also used to better understand the role of interval dosing here considered as a continuous exposure. Risks were reported as Hazard Ratios (HR) along with their 95% confidence intervals (95%). The latter analysis was performed only for MRI events but not for relapses because of small number of events.

As sensitivity analyses, cumulative hazard cumulative hazard curves comparisons and Cox model analyses were also performed after right-censoring at 40 weeks after PLC infusion.

A *P* value of <0.05 was considered significant. All analyses were performed using the R environment.

### Standard protocol approvals, registrations, and patient consents

This study was conducted using longitudinal, prospectively acquired clinical data extracted from the IMSR (https://registroitalianosm.it). The IMSR was approved by the ethical committee (EC) of the Azienda Ospedaliero-Universitaria—Policlinico of Bari (Study REGISTRO SM001, approved on August 7, 2016) and the local EC of all participating centers. All patients signed informed consent allowing the use of demographical and clinical data for research purposes.

## Results

### Fist aim: effect of COVID Italian lockdown on OCR schedule

**Fig. 2:**
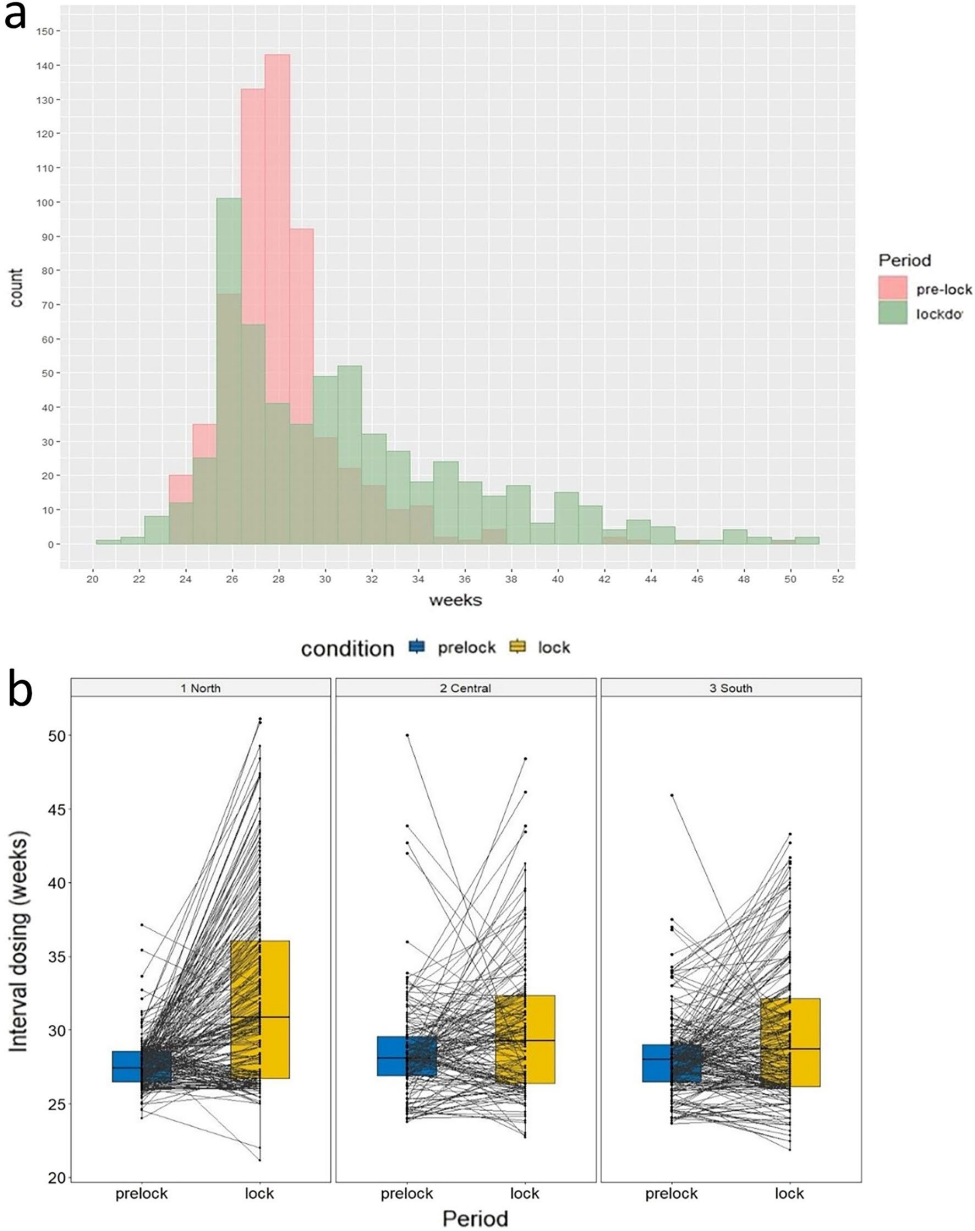
**a** Distribution of interval dosing: bar chart comparing interval between OCR infusions before (pink) and after (green) the observational period, **b** Box plots showing interval dosing (weeks) distribution before (blue) and after (yellow) the observational period in the 3 main Italian macro-regions (North, Center, South). PRE-LOCK = OCR cycle occurred between August and December 2019; LOCK = OCR cycle following pre-lock cycle, between February and June 2020

Five hundred and ninety nine pwMS (343 F/256M; 411 Relapsing MS/188 Progressive MS) from 65 MS centers were included in the analysis. Mean age was 43.93 years (Standard Deviations—SD—11.00); mean disease duration was 11.79 years (SD 8.03) (Table 1). pwMS were almost equally distributed among the three principal Italian macro-regions: 231 (38.6%) from North, 159 (26.5%) from the Center and 209 (34.9%) from the South of Italy. Mean EDSS at the time of last OCR infusion in 2019 was 4.0 (min 0.0–max 8.0). Mean interval between OCR infusions before the observation period (PLCs interval) was 28.09 weeks (SD 2.72), with 85.5% of patients (512) receiving OCR with SID. During the observation period (PLC-LC interval), mean re-dosing interval was 30.83 weeks (SD 5.45), with 49.9% of patients (299) receiving OCR with EID. Mean difference between the two groups was 2.74 weeks (SD 5.65, *p* < 0.001) (Table2; Fig. 2a).

**Table 1:** Clinico-demographic characteristics of sample, based on macroarea of origin: patients’ characteristics were reported as mean ± SD (or median and interquartile range), and frequency and percentage for continuous and categorical variables, respectively

	North (*N* = 231)	Center (*N* = 159)	South (*N* = 209)	Total (*N* = 599)
Age, years—mean (SD)	43.75 (10.80)	44.02 (11.08)	44.36 (12.18)	44.03 (11.36)
Sex (F/M)	136 (58.9%)/95 (41.1%)	94 (59.1%)/65 (40.9%)	113 (54.1%)/96 (45.9%)	343 (57.3%)/256 (42.7%)
MS phenotype (PMS/RMS)	62 (26.8%)/169 (73.2%)	37 (23.3%)/122 (76.7%)	89 (42.6%)/120 (57.4%)	188 (31.4%)/411 (68.6%)
DD, years—mean (SD)	12.26 (8.01)	11.30 (8.08)	11.63 (8.02)	11.79 (8.03)
EDSS—median (range)	4.00 (2.50, 5.88)	4.50 (2.00, 6.00)	4.50 (2.00, 6.00)	4.00 (2.00, 6.00)

*N* number, *y* years, *SD* standard deviation, *MS* multiple sclerosis, *PMS* progressive Multiple Sclerosis, *RMS* Relapsing Multiple Sclerosis, *DD* disease duration, *EDSS* expanded disability status scale

**Table 2:** Time intervals between infusions, analyzed as function of the macroarea of origin

	North (*N* = 231)	Center (*N* = 159)	South (*N* = 209)	Total (*N* = 599)
OCR-ID-PRE-LOCK (∆1), wk—mean (SD)	27.65 (1.65)	28.58 (3.59)	28.20 (2.86)	28.09 (2.73)
∆1 subgroups, *N*				
∆1 ≥ 22 and < 26	18 (7.8%)	27 (17.0%)	34 (16.3%)	79 (13.2%)
∆1 ≥ 26 and < 30	198 (85.7%)	96 (60.4%)	139 (66.5%)	433 (72.3%)
∆1 ≥ 30	15 (6.5%)	36 (22.6%)	36 (17.2%)	87 (14.5%)
OCR-ID-LOCK-PRE-LOCK (∆2), week—mean (SD)	32.32 (6.19)	30.07 (4.72)	29.75 (4.71)	30.83 (5.45)
∆2 subgroups, *N*				
∆2 ≥ 22 and < 26	13 (5.6%)	23 (14.5%)	33 (15.8%)	69 (11.5%)
∆2 ≥ 26 and < 30	81 (35.1%)	62 (39.0%)	88 (42.1%)	231 (38.6%)
∆2 ≥ = 30	137 (59.3%)	74 (46.5%)	88 (42.1%)	299 (49.9%)

*N* number; *wk* weeks; *SD* standard deviation*; OCR* Ocrelizumab*; PRE-LOCK* OCR cycle occurred between August and December 2019; *ID-PRE-LOCK* time interval, in weeks, between all the infusions prior to the pre-lock cycle; *LOCK* OCR cycle following pre-lock cycle, between February and June 2020; *ID-LOCK-PRE-LOCK* time interval, in weeks, between the pre-lock and the lock cycle

Neither demographics nor disease-related factors emerged as predictors of infusion postponement, except for the location of the MSc in the North of Italy (4.7 weeks vs. 1.5 in the Center and 1.6 in the South). Such a difference was confirmed in multivariate analysis (*p* < 0.001) adjusting for mean OCR infusion intervals during PLCs (Fig. 2b).

Considering only RMS patients (*n* = 717), 364 patients followed a SID (RMS-SID) treatment regimen, while 353 were treated with an EID strategy (RMS-EID). The two groups (RMS-SID vs. RMS-EID) did not differ in age, male/female ratio and macro-region of origin. Similarly, they did not show differences in clinical parameters with the exception concerning the number of OCR infusions before the observation period which was significantly lower in RMS-EID patients compared to RMS-SID patients (Table 3).

**Table 3:** Clinico-demographic characteristics, based on OCR interval dosing: patients’ characteristics were reported as mean ± SD (or median and interquartile range)

	RMS-SID (*n* = 364)	RMS-EID (*n* = 353)	*P*
Age, year—mean (SD)	40.59 (10.56)	42.23 (10.92)	n.s
Sex (F/M)	236/128	227/126	n.s
Macroregion (N/C/S)	132/118/114	160/113/80	n.s
DD, year—mean (SD)	10.84 (7.94)	11.56 (8.72)	n.s
EDSS—median (range)	2.5 (0–7)	3 (0–7)	n.s
Previous OCR inf—mean (SD)	2.21 (1.47)	1.78 (0.91)	**0.007**
Delta PRE-LOCK OCR inf, wk—mean (SD)	28.04 (3.75)	29.03 (4.18)	n.s

Bold: significant results

*n* number;* y* years; *wk* weeks; *SD* standard deviation; RMS relapsing multiple sclerosis; *SID* standard interval dosing; *EID* extended interval dosing; *DD* disease duration; *EDSS* expanded disability status scale; *PRE-LOCK* OCR cycle occurred between August and December 2019; *ID-PRE-LOCK* time interval, in weeks, between all the infusions prior to the pre-lock cycle; *P* Bonferroni-adjusted *P*-values

### Second aim: effect of delay on clinical/MRI activity

**Fig. 3:**
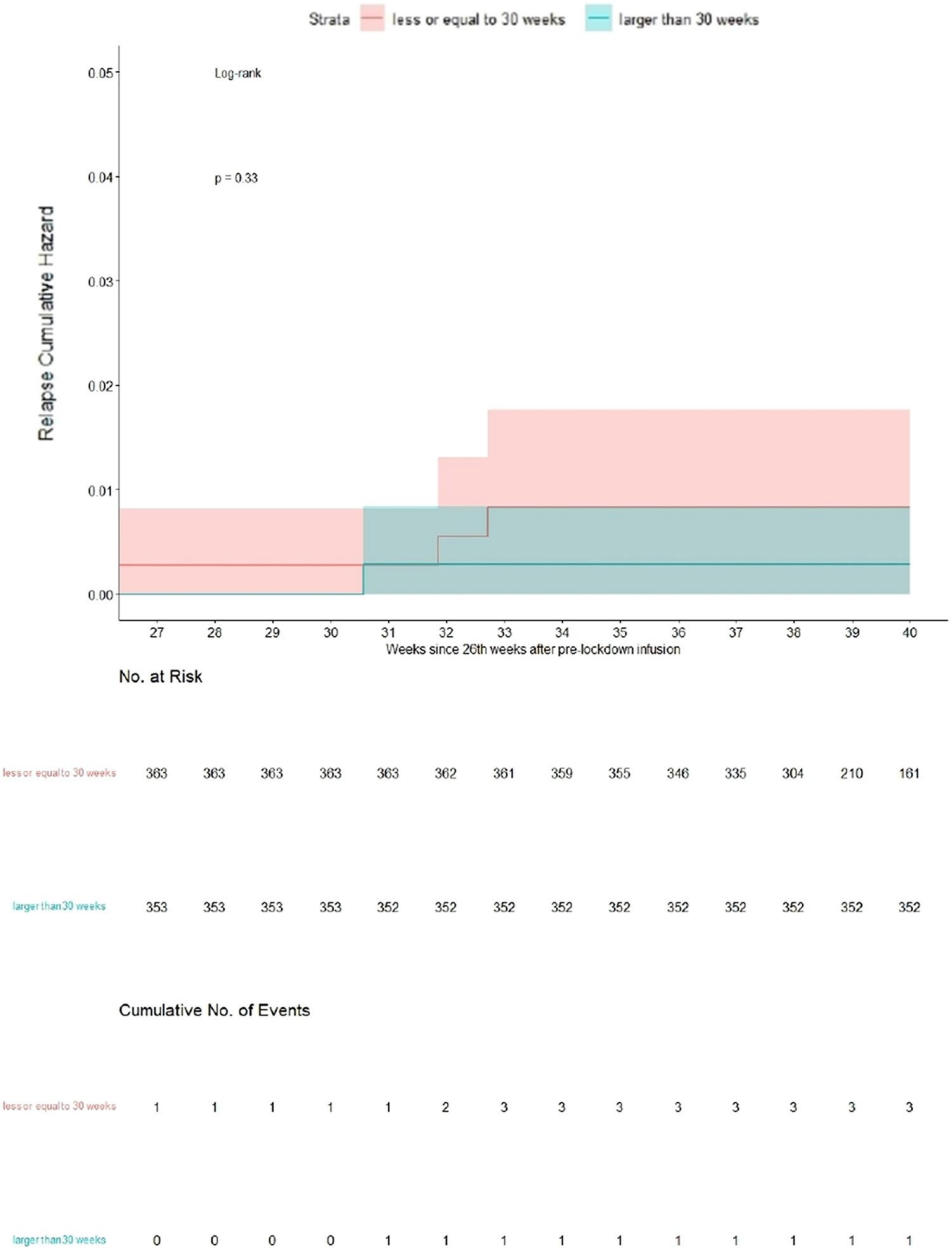
Relapses Cumulative Hazard curves, right-censored at 40 weeks after pre-lock infusion: the two populations were compared according to dichotomized interval dosing ≤ 30 weeks versus > 30 weeks, focusing the analysis within the 40th week after pre-lock infusion

Seven hundred and seventeen pwMS (364 SID/353 EID) from 65 MS centers were included in the analysis. There were 25 reports of relapses occurred after the PLC: 14 (14/717 = 1.95%) reports in RMS-SID patients and 11 (11/717 = 1.53%) in RMS-EID patients. Four RMS patients on SID regimen (4/364 = 1.1%) showed clinical activity beyond 30 weeks after the PLC: one before and three after the LC (eight weeks after retreatment). Two RMS patients in EID regimen (2/353 = 0.57%) showed clinical activity beyond 30 weeks after the PLC: one day and eight weeks after LC, respectively. The cumulative hazard for clinical relapses at 40 weeks after pre-lock infusion (i.e., 12 weeks beyond the mean OCR infusion interval in Italy before the observation/lockdown period) was < 0.02 in RMS patients receiving SID versus < 0.01 in RMS patient receiving EID (Fig. 3).

Five hundred and seventy nine total MRI reports were available in the cohort of RMS patients (414 RMS patients had at least one MRI report). There were 27 (27/364 = 7.41%) reports of MRI activity with new T2-hyperintense lesions and/or new T1-Gd + enhancing lesions in RMS-SID patients and 22 (22/353 = 6.23%) in RMS-EID patients. Four RMS patients on SID regimen (4/364 = 1.1%) showed radiological activity beyond 30 weeks after the PLC, two before and two after the LC (4 weeks after retreatment). Thirteen RMS patients on EID regimen (13/353 = 3.7%) showed radiological activity beyond 30 weeks after the PLC: seven before the LC and six after the LC.

**Fig. 4:**
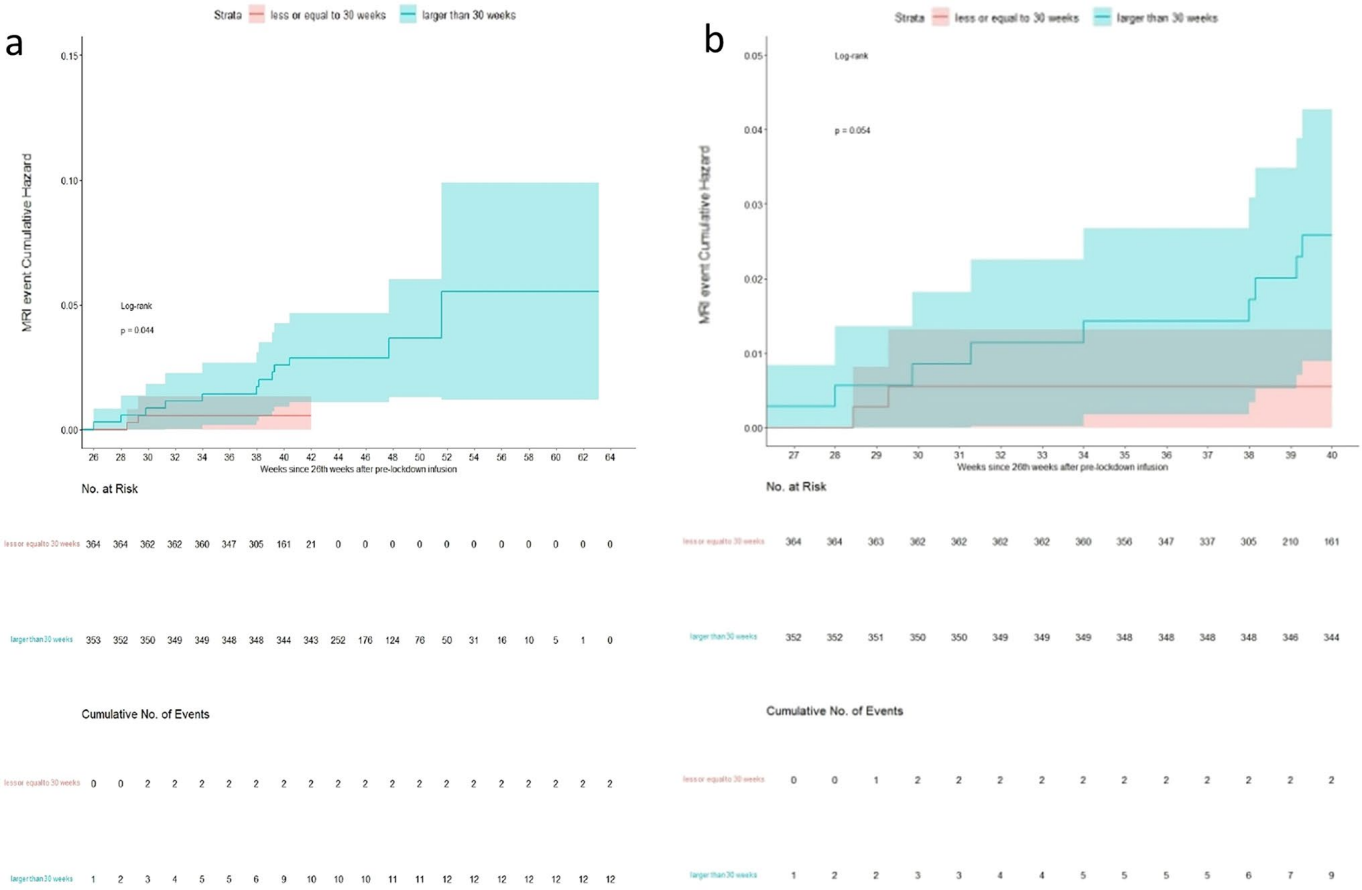
**a** MRI events Cumulative Hazard curves of two populations, compared according to the dichotomized DELTA LOCK-PRE-LOCK: patients were divided by setting a DELTA LOCK-PRE-LOCK (time interval, in weeks, between the pre-lock and the lock cycle) cut-off at 30 weeks, **b** MRI events Cumulative Hazard curves, right-censored at 40 weeks after pre-lock infusion: the two populations were compared according to dichotomized interval dosing ≤ 30 weeks versus > 30 weeks, focusing the analysis within the 40th week after pre-lock infusion

The cumulative risk for new T2-hyperintense lesions in the entire RMS population increased in a time-related fashion after week 30 from PLC and stood around 0.025 at 40 weeks after pre-lock infusion (Fig. 4a). Moreover, 1-week delay in infusion interval was associated with a 5% higher risk in developing an MRI event (HR = 1.05, 95%CI = 0.95–1.15, *p* = 0.33) although not significantly. In the censored analysis, the cumulative hazard at 40 weeks was < 0.01 in patients receiving SID and < 0.03 in patients receiving EID (Fig. 4b) and the estimated HR from Cox model did not change.

For a descriptive purpose only, in the subgroup of RMS patients treated with EID, no relapses and 7 MRI new events were reported before the LC. MRI events occurred in: 1 patients treated with a PLC-LC interval between 30 and 32 weeks (*N* = 92); 4 patients treated with a PLC-LC interval between 32 and 34 weeks (*N* = 80) and in 1 patient treated with a PLC-LC interval between 38 and 40 weeks (*N* = 25).

In view of such low clinical and radiological event rate, it was not possible to compare SID and EID outcome measures.

Due to short follow-up period and lack of uniform data collection, we couldn’t use the EDSS progression as a clinical outcome.

## Discussion

This retrospective, nation-wide, registry-based, observational study depicts the management of OCR-treated pwMS by Italian MS neurologists during the first COVID-19 outbreak that brought to a long-lasting lockdown in Italy in spring 2020.

The first observation is that in Italy, during the abovementioned period, OCR infusions were postponed on average by 2.7 weeks compared to the pre-pandemic period, with approximately half of pwMS (from the initial 14.5%) receiving an EID regimen.

This data suggested OCR schedule was mostly impacted in those areas of the country where COVID-19 pandemic hit hard and first. In fact, the sole predictor of OCR schedule delay was the location of the MSC in northern Italy, where the average OCR infusion delay was three times longer than the other macro-regions.

Moving to the second aim of our study (effect of OCR-EID on MS activity), only 6 clinical relapses and 17 MRI reports of new/enhancing lesions were recorded in the whole RMS group during the follow-up period. These results, in addition to confirming the high efficacy of OCR, support a long-lasting clinical and radiological effect of the drug as well as the lack of a rebound activity up to 40-week interval dosing. Notably, in our cohort, the cumulative risk of relapse or MRI activity was very low at 12 weeks of delay in both EID and SID cohorts and appeared to be substantially independent of the length of the infusion delay. These observations are in line with data extrapolated from pivotal and real-world studies focusing on this topic [4, 11, 13].

Of particular interest was the very low number of clinical and/or MRI events experienced by our EID cohort before re-treatment: no clinical and only 7 MRI events.

All these results are in line with most previous similar studies [9, 14, 15, 19] and with the annualized relapse and MRI events rate expected from OCR phase 3 trials (0.16 and 0.02, respectively) [10], confirming that OCR infusion delay does not compromise its short-term efficacy.

In contrast to our results, a recent retrospective Italian study [20] found that EID was associated with a significantly increased MRI activity. This discrepancy with our data might be explained by: (1) a smaller sample size; (2) a longer observation time that extended until June 2021, such that included a long period outside the lockdown, thus impacting data collection in EID patients. All abovementioned reasons could at least partly explain the surprisingly high number of radiological and—most of all—clinical activity observed in both groups, paradoxically higher in the SID-treated patients. Notably, 24 relapses were observed in 18 patients, thus reporting an unexpectedly high clinical activity when compared with previous pivotal and real-world OCR studies. Moreover, MRI activity was exceedingly low [17] with only two relapsing patients showing concomitant MRI activity. Finally, it must be emphasized that authors conclusions relied only on the results of a multivariable model. In fact, as in our results, no significant differences were observed between the SID and EID cohorts in terms of change in NEDA-3 status.

### Limitations

First, being registry-based, our study could have been affected by incomplete or incorrect data entry. A careful preliminary check and cleaning of the database was made and followed—when needed—by requests to participating MSC to revise/complete incomplete/incongruent/missing data, thus limiting database inconsistences, but still many patients could not be included in the analysis for missing/incomplete data.

Second, the mean interval of dosing in the SID group is 28.1 weeks, versus 30.8 weeks in the EID; this makes a delay of 2.7 weeks. Although this is statistically significant, it is a very little difference. This may be a shortcoming of the study leading to a weakened statistical power.

Third, it is worthy to underpin that the shortness of the follow-up period prevented the evaluation of EDSS in RRMS and progressive pwMS.

Fourth, the present study does not take into account CD19/CD20 count immunoglobulin levels, due to the non-mandatory nature of this data in the IMSR.

Finally, the low relapse and MRI activity recorded in both (SID and EID) group and the immortal time bias implied in clinical and radiological disease activity, although emphasizing the efficacy of OCR, limit the statistical power of the study.

### Conclusions

To our knowledge, this is the first nation-wide, registry-based, observational study, that explored the effect of OCR SID versus EID in a large and unbiased pwMS sample, using the disruption of OCR infusion schedule that occurred during the first Italian COVID-19 lockdown.

At that time, OCR infusions were significantly delayed in Italy, in particular in the northern regions, thus reflecting both clinicians concerns about using an immunosuppressive DMT during the threats of a new pandemic virus, and the disruption of the national health system [2], with significant reallocation of healthcare resources.

Despite the significant extension of OCR infusion intervals, the incidence of clinical and MRI events was very low up until 12 weeks of follow-up (i.e., 40-week interval dosing), thus suggesting durable efficacy of OCR, as well as the absence of rebound after its short-term suspension.

To further corroborate and expand these findings, other predictors/outcome parameters, such as standardized CD20 B lymphocytes count and clinical disability measures (such EDSS), will need to be assessed in future studies with a longer follow-up.

Despite a number of limitations, our study suggest that the extension of OCR infusion intervals beyond 30 weeks and up to 40 weeks could represent an option for MS neurologists concerned by the risk–benefit ratio of this immunosuppressive treatment in particular situations such a virus outbreak/pandemic, a vaccine campaign or other comorbidities/conditions that might enhance the risk of opportunistic infections or other drug-related adverse events.

## Data Availability

Not applicable.
